# Supplementary material for: Integrating isoniazid preventive therapy into the fast-track HIV treatment model in urban Zambia: A proof-of -concept pilot project
Source: PLOS Glob Public Health. 2023 Mar 8;3(3):e0000909. doi: 10.1371/journal.pgph.0000909 (PMC10021523; doi:10.1371/journal.pgph.0000909)
Supplement: S1 Table — (DOCX) [file pgph.0000909.s001.docx]

***S1 Table: Sensitivity analysis of participants who kept/did not keep all phone appointments and participants with /without phones.***

| **Characteristics** |  | **Number (%) Kept All Appointments** | | **P-value** | **Number (%) With phone** | | **P-value** | |
| --- | --- | --- | --- | --- | --- | --- | --- | --- |
| **Age** |  | **Yes** | **No** |  | **Yes** | **No** |  |  |
| <30 | 89 (10.9%) | 60 (67.4%) | 29 (32.6%) | 0.9 | 89 (10.9%) | 23  (12.9%) | 0.7 |  |
| 30-39 | 270 (33%) | 174 (64.4%) | 96 (35.6%) |  | 270 (33%) | 52 (29.2%) |  |  |
| 40-49 | 286 (35%) | 192 (67.1%) | 94 (32.9%) |  | 286 (35%) | 62 (34.8%) |  |  |
| 50+ | 173 (21.15%) | 113(65.3%) | 60 (34.7%) |  | 173 (21.2%) | 41 (23%) |  |  |
| **Sex** |  |  |  | 0.8 |  |  | **0.001** |  |
| Female | 540 (66%) | 354 (65.6%) | 186 (34.4%) |  | 540 (66%) | 141 (79.2%) |  |  |
| Male | 278 (34%) | 185 (66.6%) | 93 (33.4%) |  | 278 (34%) | 37 (20.8%) |  |  |

***Data Analysis***

[analysis_dataset.xlsx](https://cidrzit-my.sharepoint.com/:x:/g/personal/paul_somwe_cidrz_org/ERvQMlT9IshDlP8PbRW36DsBxgmg1A8lDLse8HcjqoeN9Q?e=FcjcKe)
