# Supplementary material for: Integrating isoniazid preventive therapy into the fast-track HIV treatment model in urban Zambia: A proof-of -concept pilot project
Source: PLOS Glob Public Health. 2023 Mar 8;3(3):e0000909. doi: 10.1371/journal.pgph.0000909 (PMC10021523; doi:10.1371/journal.pgph.0000909)
Supplement: S1 Text — (PDF) [file pgph.0000909.s004.pdf]

Selfcare with Tuberculosis Prevention Therapy Project
Tuberculosis screening and adverse drug reaction/side effects Exit Form

Date:_________________________

ART Number:____ **Gender:**____________________

Section A Rapid TB symptom

Please ask the patient if they have experienced any of these **symptoms**. Please indicate the response for **each** symptom by checking **'Yes'** or **'No'** below. The duration or quality of symptoms does not matter. All that matters is whether the patient reports the symptom.

	Yes	No
1. Current cough?	☐	☐
2. Fever?	☐	☐
3. Night sweats?	☐	☐
4. Weight loss?	☐	☐

If the patient answered **'Yes'** to ***ANY*** of **the** above, take the patient to the clinician for evaluation

Section B: Side effects
Side effects of IPT

Did you experience any of the following side effects in the last 4 weeks?

- ❖ Common in the first month of treatment

	Yes	No
1. Burning sensation in the fingers and toes	☐	☐
2. Itchy skin, yellow eyes, tongue and palms	☐	☐
3. Joint pains	☐	☐
4. Headache	☐	☐
5. Diarrhea	☐	☐
6. Nausea	☐	☐
7. Stomach pains	☐	☐
8. Increased appetite	☐	☐
9. Vomiting	☐	☐

If the patient answered **'Yes'** to ***ANY*** of the above, the add the list to the ADR list and hand it the coordinator to complete the pharmacovigilance form

Section C: Adherence & Challenges faced taking drugs

1. Are you still taking your IPT? ☐ Yes ☐ No
2. When did you last take your IPT?__________________ (Number of days ago)
3. Are there times you forgot to take your IPT? ☐ Yes ☐ No
4. If yes reasons for missing IPT?
    a. Forgot ☐Yes ☐ No
    b. Left the drugs at home ☐ Yes ☐ No
    c. Side effects ☐Yes ☐ No
    d. Other specify:____
5. How many pills do have remaining? _______________
6. What time do you take your pills? ________________

Section D Outcomes

Please mark all that apply

Completed Treatment	☐	Died	☐
Stopped against Medical advice	☐	LTFU	☐
Not evaluated	☐	Developed Active TB	☐

Comments:____

____

____

____

____
