# Supplementary material for: Integrating isoniazid preventive therapy into the fast-track HIV treatment model in urban Zambia: A proof-of -concept pilot project
Source: PLOS Glob Public Health. 2023 Mar 8;3(3):e0000909. doi: 10.1371/journal.pgph.0000909 (PMC10021523; doi:10.1371/journal.pgph.0000909)
Supplement: S2 Text — (PDF) [file pgph.0000909.s005.pdf]

Selfcare with Tuberculosis Prevention Therapy Project
Tuberculosis screening and adverse drug reaction/side effects Follow-up Form

Date:____

Patient Number:_____________________________________ **Gender:**__________________

Section A Rapid TB symptom

Please ask the patient if they have experienced any of these **symptoms**. Please indicate the response for **each** symptom by checking **'Yes'** or **'No'** below. The duration or quality of symptoms does not matter. All that matters is whether the patient reports the symptom.

	Yes	No
1. Current cough?	☐	☐
2. Fever?	☐	☐
3. Night sweats?	☐	☐
4. Weight loss?	☐	☐

Section B: Side effects

Side effects of IPT

Did you experience any of the following side effects in the last 4 weeks?

- Common in the first month of treatment

	Yes	No
1. Burning sensation in the fingers and toes	☐	☐
2. Itchy skin, yellow eyes, tongue and palms	☐	☐
3. Joint pains	☐	☐

Ask the client to return to the clinic if the clinic if they reported any symptoms 1-3 with 24 hours

	Yes	No
4. Headache	☐	☐
5. Diarrhea	☐	☐
6. Nausea	☐	☐
7. Stomach pains	☐	☐
8. Increased appetite	☐	☐
9. Vomiting	☐	☐

If the patient answered **'Yes'** to ***ANY*** of the above, the add the list to the ADR list and hand it the coordinator to complete the pharmacovigilance form

Section C: Adherence & Challenges faced taking drugs

1. Are you still taking your IPT? ☐ Yes ☐ No

2. When did you last take your IPT?__________________ (Number of days ago)

3. Are there times you forgot to take your IPT? ☐ Yes ☐ No

4. If yes reasons for missing IPT?
   a. Forgot ☐ Yes ☐ No
   b. Left the drugs at home ☐ Yes ☐ No
   c. Side effects ☐ Yes ☐ No
   d. Other specify:____

5. How many pills do have remaining? _______________

6. What time do you take your pills? ________________

7. Comments:____

   ____

   ____

   ____

   ____
