# Supplementary material for: Integrating isoniazid preventive therapy into the fast-track HIV treatment model in urban Zambia: A proof-of -concept pilot project
Source: PLOS Glob Public Health. 2023 Mar 8;3(3):e0000909. doi: 10.1371/journal.pgph.0000909 (PMC10021523; doi:10.1371/journal.pgph.0000909)
Supplement: S3 Text — (PDF) [file pgph.0000909.s006.pdf]

Selfcare with Tuberculosis Prevention Therapy Project
Tuberculosis screening and adverse drug reaction/side effects Enrollment Form

Date:____

Patient Number:_______________________________________ **Gender:**____________________

Section A Rapid TB symptom

Please ask the patient if they have experienced any of these **symptoms**. Please indicate the response for **each** symptom by checking **'Yes'** or **'No'** below. The duration or quality of symptoms does not matter. All that matters is whether the patient reports the symptom.

	Yes	No
1. Current cough?	☐	☐
2. Fever?	☐	☐
3. Night sweats?	☐	☐
4. Weight loss?	☐	☐

If the patient answered **'Yes'** to ***ANY*** of the above, Patient is not eligible for TPT. Send patient for sputum collection

Section B: Enrollment Status

1. Has patient been enrolled in the study? Yes ☐ No ☐
2. If no, reasons for not enrolling in the study (Please mark all that apply)
   a. Not willing to participate in the study Yes ☐ No ☐
   b. Active TB Yes ☐ No ☐
   c. Recently completed IPT Yes ☐ No ☐
   d. Pregnant Yes ☐ No ☐
   e. Patient does not have a phone Yes ☐ No ☐
   f. Other specify:____
