# Supplementary material for: Integrating isoniazid preventive therapy into the fast-track HIV treatment model in urban Zambia: A proof-of -concept pilot project
Source: PLOS Glob Public Health. 2023 Mar 8;3(3):e0000909. doi: 10.1371/journal.pgph.0000909 (PMC10021523; doi:10.1371/journal.pgph.0000909)
Supplement: Attachment — Submitted filename: PLOSGPH Response to Reviewers_30Dec22.docx [file pgph.0000909.s007.docx]

Sabine Hermans
Academic Editor
PLOS Global Public Health

Journal Requirements:

1. Please amend your detailed Financial Disclosure statement. This is published with the article. It must therefore be completed in full sentences and contain the exact wording you wish to be published.

a. State what role the funders took in the study. If the funders had no role in your study, please state: “The funders had no role in study design, data collection and analysis, decision to publish, or preparation of the manuscript.”
b. If any authors received a salary from any of your funders, please state which authors and which funders.

**Response:** The Financial Disclosure statement has been amended as follows “*The funder had no role in study design, data collection and analysis, decision to publish, or preparation of the manuscript. Furthermore, none of the authors received a salary from the funders”* ***page 27***

2. In the online submission form, you indicated that "The Government of Zambia allows data sharing when applicable local conditions are satisfied. In this case, the data from the study will be made available to any interested researchers upon request. The CIDRZ Ethics and Compliance Committee is responsible for approving such request. To request data access, one must write to the Committee chair/Chief Scientific Officer, Dr. Roma Chilengi, ([Roma.Chilengi@cidrz.org](mailto:Roma.Chilengi@cidrz.org)) or the Secretary to the Committee/Head of Research Operations, Ms. Hope Mwanyungwi ([Hope.Mwanyungwi@cidrz.org](mailto:Hope.Mwanyungwi@cidrz.org)) mentioning the intended use for the data. The Committee will then facilitate review and authorization to release the data as requested. Data requests must include contact information, a research project title, and a description of the intended use.". All PLOS journals now require all data underlying the findings described in their manuscript to be freely available to other researchers, either 1. In a public repository, 2. Within the manuscript itself, or 3. Uploaded as supplementary information.
This policy applies to all data except where public deposition would breach compliance with the protocol approved by your research ethics board. If your data cannot be made publicly available for ethical or legal reasons (e.g., public availability would compromise patient privacy), please explain your reasons by return email and your exemption request will be escalated to the editor for approval. Your exemption request will be handled independently and will not hold up the peer review process but will need to be resolved should your manuscript be accepted for publication. One of the Editorial team will then be in touch if there are any issues.

**Response:** We have uploaded the data and tools as supplementary information.

Additional Editor Comments (if provided):


[Note: HTML markup is below. Please do not edit.]

Reviewers' comments:

Reviewer's Responses to Questions

**Comments to the Author**

1. Does this manuscript meet PLOS Global Public Health’s [publication criteria](https://eur03.safelinks.protection.outlook.com/?url=https%3A%2F%2Fjournals.plos.org%2Fglobalpublichealth%2Fs%2Fcriteria-for-publication&data=05%7C01%7Cmpande.mwenechanya%40cidrz.org%7C728e4d9cf34c4996fc9c08dac89e28ce%7C389f1ebbd7774ab58def42a5441fb23d%7C0%7C0%7C638042878859955165%7CUnknown%7CTWFpbGZsb3d8eyJWIjoiMC4wLjAwMDAiLCJQIjoiV2luMzIiLCJBTiI6Ik1haWwiLCJXVCI6Mn0%3D%7C3000%7C%7C%7C&sdata=9SAj0vknqrgZ4U7wy87RuIHKZTGmDBefK03%2Fr1nsLbg%3D&reserved=0)? Is the manuscript technically sound, and do the data support the conclusions? The manuscript must describe methodologically and ethically rigorous research with conclusions that are appropriately drawn based on the data presented.

Reviewer #1: Partly

Reviewer #2: Yes

2. Has the statistical analysis been performed appropriately and rigorously?

Reviewer #1: Yes

Reviewer #2: No

3. Have the authors made all data underlying the findings in their manuscript fully available (please refer to the Data Availability Statement at the start of the manuscript PDF file)?

The [PLOS Data policy](https://eur03.safelinks.protection.outlook.com/?url=https%3A%2F%2Fjournals.plos.org%2Fglobalpublichealth%2Fs%2Fdata-availability&data=05%7C01%7Cmpande.mwenechanya%40cidrz.org%7C728e4d9cf34c4996fc9c08dac89e28ce%7C389f1ebbd7774ab58def42a5441fb23d%7C0%7C0%7C638042878859955165%7CUnknown%7CTWFpbGZsb3d8eyJWIjoiMC4wLjAwMDAiLCJQIjoiV2luMzIiLCJBTiI6Ik1haWwiLCJXVCI6Mn0%3D%7C3000%7C%7C%7C&sdata=8LjLSwAL5ZQHIZPq5mF0GVOZInhx6TN0MnsCWPxVWE0%3D&reserved=0) requires authors to make all data underlying the findings described in their manuscript fully available without restriction, with rare exception. The data should be provided as part of the manuscript or its supporting information, or deposited to a public repository. For example, in addition to summary statistics, the data points behind means, medians and variance measures should be available. If there are restrictions on publicly sharing data—e.g. participant privacy or use of data from a third party—those must be specified.

Reviewer #1: No

Reviewer #2: No

4. Is the manuscript presented in an intelligible fashion and written in standard English?

PLOS Global Public Health does not copyedit accepted manuscripts, so the language in submitted articles must be clear, correct, and unambiguous. Any typographical or grammatical errors should be corrected at revision, so please note any specific errors here.

Reviewer #1: Yes

Reviewer #2: Yes

5. Review Comments to the Author

Please use the space provided to explain your answers to the questions above. You may also include additional comments for the author, including concerns about dual publication, research ethics, or publication ethics. (Please upload your review as an attachment if it exceeds 20,000 characters)

Reviewer #1: Major concerns

1. A major concern is the way in which the lessons learnt and challenges were captured. More information is needed on whether a structured analytical approach was followed, for instance a thematic analysis to “… synthesize key lessons learned…” or in which alternative way the “… implementers, facility-based clinicians and community health workers assessed the tools and processes to be effective and relatively efficient.” (lines 203-233)

**Response:** Thank you for the feedback. The lessons learned were discussed in monthly meetings and documented in monthly meeting notes. We read and re-read all monthly meeting notes to identify and abstract all text on lessons learned during implementation including contextual factors (enablers and hinderances) and attendees’ reflections on their own actions and relationships. We reviewed/ discussed conclusions with the implementation team. We have described this approach at the end of the Methods section **lines 110-116,** Additionally, we have a description of the project purpose to the introduction which we hope explains the connection: “*Objectives included documenting the inputs, processes and enabling factors and challenges involved in launching this new approach to TPT delivery, as well as client uptake, completion of in-person and phone check-ins, and self-reported TPT completion rates” (****line 66****).* We report and recommend that the lack of structured analytical approach to report lessons learnt as a limitation and recommend that future studies utilize a thematic/realist analysis to synthesize key lessons learned (**lines 281-285**).

2. Second, adherence and side effects were measured through self-report. Did the authors consider an alternative approach to measure adherence, for instance 99DOTS or measuring drug levels through a urinary assay? Self-reported adherence is notoriously overestimated. The authors might want to include this as a suggestion for future studies. For side effects, a telemedicine approach might be valuable.

**Response:** We agree with the reviewers that reliance on self-report to assess adherence may lead to overestimates; however, budgetary constraints did not allow for alternative approaches to measure adherence. We have included the recommendation to use alternative approaches to measure adherence, for instance 99DOTS or measuring drug levels through a urinary assay in future studies (**line 285**). For side effects monitoring we prospectively collected at every phone call, we believe that this innovation of provision of IPT long refills and utilization of phone calls in between clinic visits is an intervention that may be leveraged to support uptake and completion of other TPT. We think this as strength of the pilot and have included this in the discussion (**lines 273-277***).*

3. Third, did the authors consider a sensitivity analysis to look at the potential participants without phones who were excluded (15%). A selection bias might have been introduced if these potential participants were substantially different from their peers, which might have been the case particularly on a socio-economic level if they did not have phones. It would also be interesting to know whether the participants who could not be found at home visits (25%), were different from their peers.

**Response:** Thank you for comment, the sensitivity analysis was conducted to assess differences in participants who kept all phone appointments and those who did not and hence visited at their homes. Furthermore, we looked at differences of those with phone vs without phones at enrollment. The analysis showed insignificant differences across most thus, we did not include this in main paper. However, we have added the sensitivity analysis results as supplementary information (***line 305***). Whilst, more females than males were recruited in the study, the females were mostly likely not to have phones at enrollment.

4. Finally, the reported IPT adherence in the project was 90.2% vs. the national completion rate of 90%. Since there is no apparent statistical significance between 90.2% and 90%, what would the added value be when this pilot is scaled-up? I would suggest an additional emphasis on uptake of IPT, which was significantly higher than in the standard of care (>70% vs. <50%).

**Response:** We agree with the reviewers that high IPT uptake reported in this study was an additional pilot value thus in the discussion we report as follows “*Uptake of IPT amongst PLHIV in Zambia ranges from 18% to 49% while in this pilot 70.1% of all enrolled participants accepted the intervention.^4,31^ We hypothesize that the person-centered and convenient model being piloted led to higher than usual 70.1% uptake rate, without detriment to the completion rate (90.2% to standard of care 90%). DSD models that extended clinic visits have shown similar uptake success in HIV programs ^32-34^ thus, supporting client’s preference for extended integrated visits.*” (**lines 254-257**) Additionally, the combination of provision of long refills of IPT and access to ongoing phone-based counselling and support is an innovative strategy to facilitate social support (**line 275**).

Minor concerns

1. Line 84: please clarify this sentence “We believed this site to be relatively common in our country (even if not typical) to quickly learn and generate valuable lessons…” – my assumption is that the authors wanted to make the point that the site experience could be generalized in a way.

**Response:** Thank you for this, the sentence was made to provide clarity that this proof-of-concept pilot was conducted under the auspices of the Ministry of Health and show that that the setting was relatively common. The intention was not to assert any generalizability, merely show that the findings contribute to the dialogue about integration of TPT into differentiated HIV treatment models. Furthermore, we have taken care that our language throughout the manuscript does not to overstate our findings or their generalizability and state the single site as limitation in the discussion (**line 280**)

2. Please clarify in Figure 3, if a HIV clinic visit/ART pick-up was conducted at three months anyway, why would participants still need to be called for IPT? Should the HIV clinic visit not be scheduled at 6 months?

**Response:** The intervention was adapted for integration into the Fast Model which is “an individual facility-based model in which clients visit health facilities every three months, alternating expedited medication pick up visits and clinical visits as shown in (Figure 1) (**page 6**). Therefore, participants that came through at 3 months were assessed in-person and did not receive a phone call.

3. Please check Table 1, the last column does not add up (Sex and ART duration).

**Response:** Thankyou table 1 has been revised accordingly (**page 15**)

Reviewer #2: General comment: Relevant topic regarding delivery of TB Preventive Therapy (TPT) in the era of Differentiated Service Delivery (DSD).
1. One of the most worried things in TPT is Breakthrough TB diseases. Hence, WHO recommends regular screening of TB using symptoms and screening. There is no TB screening in the piloted model. Was it done?
I think for the model to function well, there should be a way to monitor development of TB during TPT. Continuation of TPT in the presence of TB diseases leads to inappropriate treatment which may lead to the development of drug resistance mutation

**Response:** We agree with the reviewer on WHO recommendations for regular screening of TB symptoms for participants on TPT. It is for this reason that in this pilot we ensured that TB screening was conducted at each interaction with the participant either by phone/physical (**Pages 7, 10 11, lines 107, 119** ).

2. The authors mentioned about using Fishers exact test to determine the association between outcomes and exposures. However, there were not 2 by 2 shown despite stating that there were no statistically significant differences.
By virtue of biological plausibility or previous literature, one would go further to carry multivariable regression analysis to determine the relationship.

**Response:** Thank you for this feedback. We have revised the table accordingly. The reviewer is correct that by virtue of biological plausibility the univariable analysis should be taken further to multivariable analysis however, we did not proceed as none of the variables were associated with p-value <0.005.

6. PLOS authors have the option to publish the peer review history of their article ([what does this mean?](https://eur03.safelinks.protection.outlook.com/?url=https%3A%2F%2Fjournals.plos.org%2Fglobalpublichealth%2Fs%2Feditorial-and-peer-review-process%23loc-peer-review-history&data=05%7C01%7Cmpande.mwenechanya%40cidrz.org%7C728e4d9cf34c4996fc9c08dac89e28ce%7C389f1ebbd7774ab58def42a5441fb23d%7C0%7C0%7C638042878859955165%7CUnknown%7CTWFpbGZsb3d8eyJWIjoiMC4wLjAwMDAiLCJQIjoiV2luMzIiLCJBTiI6Ik1haWwiLCJXVCI6Mn0%3D%7C3000%7C%7C%7C&sdata=zyRILroAYlm25EiMHN1daDbYrEVuIn3MmBwKPFABM%2Fk%3D&reserved=0)). If published, this will include your full peer review and any attached files.


**Do you want your identity to be public for this peer review?** If you choose “no”, your identity will remain anonymous but your review may still be made public.


For information about this choice, including consent withdrawal, please see our [Privacy Policy](https://eur03.safelinks.protection.outlook.com/?url=https%3A%2F%2Fwww.plos.org%2Fprivacy-policy&data=05%7C01%7Cmpande.mwenechanya%40cidrz.org%7C728e4d9cf34c4996fc9c08dac89e28ce%7C389f1ebbd7774ab58def42a5441fb23d%7C0%7C0%7C638042878859955165%7CUnknown%7CTWFpbGZsb3d8eyJWIjoiMC4wLjAwMDAiLCJQIjoiV2luMzIiLCJBTiI6Ik1haWwiLCJXVCI6Mn0%3D%7C3000%7C%7C%7C&sdata=ugkridrDzVDCRw2ncmhjTDac0EUXaFiEf%2FR8H4QXo4M%3D&reserved=0).

Reviewer #1: No

Reviewer #2: No
